# Effect of Primary Tumor Location on Second- or Later-Line Treatment With Anti-Epidermal Growth Factor Receptor Antibodies in Patients With Metastatic Colorectal Cancer: A Retrospective Multi-Center Study

**DOI:** 10.3389/fonc.2022.813009

**Published:** 2022-02-15

**Authors:** Anita Archwamety, Nattaya Teeyapun, Teerada Siripoon, Naravat Poungvarin, Suebpong Tanasanvimon, Ekaphop Sirachainan, Charuwan Akewanlop, Krittiya Korphaisarn

**Affiliations:** ^1^Division of Medical Oncology, Department of Medicine, Faculty of Medicine Siriraj Hospital, Mahidol University, Bangkok, Thailand; ^2^Division of Medical Oncology, Department of Medicine, Faculty of Medicine, Chulalongkorn University, Bangkok, Thailand; ^3^Medical Oncology Unit, Department of Medicine, Faculty of Medicine Ramathibodi Hospital, Mahidol University, Bangkok, Thailand; ^4^Department of Clinical Pathology, Faculty of Medicine Siriraj Hospital, Mahidol University, Bangkok, Thailand

**Keywords:** primary tumor location, later-line treatment, anti-epidermal growth factor receptor (EGFR) antibodies, metastatic colorectal cancer, sidedness

## Abstract

**Background:**

Current guidelines recommend anti-epidermal growth factor receptor monoclonal antibodies (anti-EGFR Ab) as first-line treatment only in patients with left-sided *RAS* wild type *(RASwt*) metastatic colorectal cancer (mCRC). However, there are no guideline recommendations specific to tumor sidedness in subsequent-line treatment. This study aimed to investigate the effect of primary tumor location on second- or later-line treatment outcomes in patients with *KRASwt* mCRC.

**Methods:**

Medical records of patients diagnosed with mCRC at 3 academic centers in Thailand (Siriraj, Chulalongkorn, and Ramathibodi hospital) between 2008 and 2019 were retrospectively reviewed. Patients with *KRASwt* mCRC who received anti-EGFR Ab in second- or later-line treatment were included. The impact of tumor sidedness on progression-free survival (PFS) was determined using Kaplan-Meier method, and those results were compared using log-rank test.

**Results:**

Among the 2,102 patients who had *KRAS* analysis data, 1,130 (54%) patients had *KRASwt*. Of those, 413 patients received anti-EGFR Ab in second- or later-line treatment. One hundred and sixty-two of 413 (39%) patients had extended *RAS* analysis. Seventy (17%) patients had right-sided tumors. Two hundred and thirty-eight (58%) patients received anti-EGFR Ab in the third line, and 132 (32%) patients and 43 (10%) patients were treated in the second and more than third line, respectively. Single-agent irinotecan was the most commonly used backbone chemotherapy (303/413, 73%). Patients with right-sided tumors had non-significantly inferior PFS compared to patients with left-sided tumors (median PFS: 5.7 months (mo), 95% confidence interval [CI]: 3.9-7.5 vs. 7.5 mo, 95% CI 6.5-8.5; p=0.17). Subgroup analysis showed no difference in PFS when stratified by treatment lines. Patient with right-sided tumors had significantly inferior OS compared to patients with left-sided tumors (median OS: 23.3 mo vs. 29.9 mo; p=0.005).

**Conclusions:**

To date, this is the largest real world data of the effect of primary tumor location on anti-EGFR Ab which demonstrated that tumor sidedness has no significant impact on treatment outcomes in *KRASwt* mCRC patients receiving second- or later-line therapy. Our findings do not support the utility of tumor sidedness for treatment selection in these settings. We confirmed that patients with right-sided tumors had significantly worse survival.

## Introduction

Colorectal cancer (CRC) is the leading cause of cancer-related death worldwide with over 1.93 million new cases and 935,000 deaths in 2020 (1). However, due to recent advancements in targeted biological therapy, the median survival duration now exceeds 30 months in patients with metastatic CRC (mCRC) (2). The epidermal growth factor receptor (EGFR), which is a transmembrane receptor tyrosine kinase that is overexpressed in several human cancers, has become an important therapeutic target in mCRC. Anti-EGFR monoclonal antibodies (anti-EGFR Ab), including cetuximab and panitumumab, are widely used in mCRC treatment, and *RAS* mutations are a negative predictive marker of anti-EGFR Ab response (3).

Retrospective analyses of data from several randomized studies have assessed the clinical effect of anti-EGFR Ab in patients with mCRC according to the location of the primary tumor (2, 4–6). The results of those analyses revealed better survival outcomes after treatment with anti-EGFR Ab plus chemotherapy versus chemotherapy alone or combined with bevacizumab in patients with left-sided mCRC. In contrast, patients with right-sided tumors generally appeared to benefit more from chemotherapy combined with bevacizumab.

The current guideline of the European Society for Medical Oncology (ESMO) (7) recommends anti-EGFR Ab as a first-line treatment for patients with left-sided *RASwt* mCRC. However, there are no recommendations specific to tumor sidedness relative to subsequent-line treatments in *RASwt* mCRC patients. Accordingly, the aim of this study was to investigate the effect of primary tumor location on second- or later-line treatment outcomes in patients with *KRASwt* mCRC.

## Materials and Methods

This multi-center, retrospective cohort study evaluated patients diagnosed with primary colorectal adenocarcinoma between 1 January 2008 and 31 December 2019 at 3 academic centers in Thailand (Faculty of Medicine Siriraj Hospital, Faculty of Medicine Chulalongkorn University hospital, and Faculty of Medicine Ramathibodi hospital, Bangkok, Thailand). We excluded patients with missing data, no histopathological data, no anti-EGFR treatment, and no treatment and follow-up at our centers. The study protocol was approved by the Siriraj Institutional Review Board (SIRB) EC3-356/2562, Med Chula IRB 664/62), MURA 2020/1287. This retrospective nature of this study ensures total anonymity of patient data, and patient health and wellbeing are in no way affected, so the requirement to obtain written informed consent from study participants was waived.

The primary objective of this study was to assess the effect of tumor location on progression-free survival (PFS) in *KRASwt* mCRC patients treated with anti-EGFR Ab as second- or later-line treatment. The secondary objective was to evaluate the impact of any influence of tumor sidedness on overall survival (OS) and objective response rate (ORR).

### Clinical Characteristics

Demographic and clinical characteristics including age, gender, primary tumor site, line of treatment, *RAS* status, microsatellite instability (MSI) status, molecular analysis technique, chemotherapy regimen used, date of diagnosis of stage IV disease, date of disease recurrence, date of starting anti-EGFR Ab, date of stopping anti-EGFR Ab, reason for stopping anti-EGFR Ab, date of last follow-up, and date of death were collected from a review of patient electronic medical records. Disease staging was determined according to the American Joint Committee on Cancer (AJCC) TNM Staging Classification System for Colon Cancer 8^th^ edition (8).

### Assessment of Primary Tumor Location

All patients with *KRASwt* mCRC who received anti-EGFR Ab as second- or later-line treatment were classified into two groups according to primary tumor location. Primary tumors located in the cecum to the splenic flexure were classified as right-sided. Tumors located from the descending colon to the rectum were categorized as left-sided.

### *RAS* Status Assessment

*RAS* analyses were performed at our centers or at local molecular analysis centers using Sanger sequencing, TheraScreen^®^ (Qiagen, Hilden, Germany), real-time polymerase chain reaction (PCR) techniques, and next-generation sequencing (NGS). Extended panel *RAS* analysis included mutations in *KRAS* exons 2, 3, and 4, and in *NRAS* exons 2, 3, and 4.

### Statistical Analysis

PFS was calculated from the date of starting anti-EGFR Ab treatment to the date of disease progression according to Response Evaluation Criteria in Solid Tumors (RECIST) version 1.1 or death (whichever occurred first). OS was calculated from the date of diagnosis of stage IV disease to the date of death from any cause.

Patient demographic and clinical characteristics were summarized descriptively. Median and range values were used for continuous variables, and frequency and percentage values were used for categorical variables. Pearson’s chi-square test or Fisher’s exact test was applied to evaluate associations between right-/left-sided tumor and clinicopathological variables. Patient survival outcome was analyzed using Kaplan-Meier method. Differences between curves were determined using log-rank test. Cox regression analysis was used to estimate the 95% confidence intervals (CIs) for PFS. A *p*-value ≤ 0.05 was the determiner of statistical significance. All analyses were performed using SPSS version 26.0 statistical software (SPSS, Inc.; IBM Corporation, Armonk, NY, USA).

## Results

**Figure 1:**
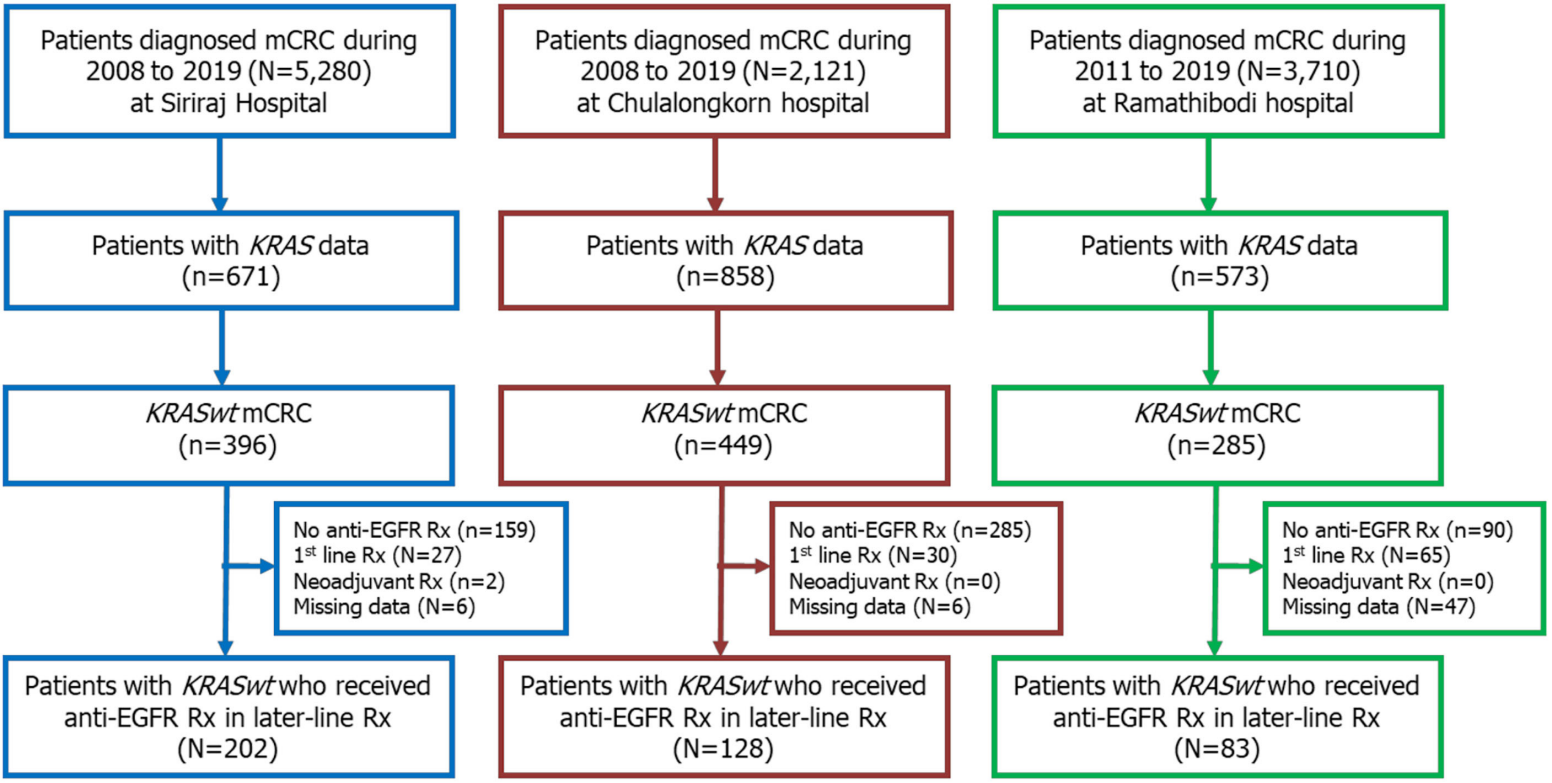
CONSORT diagram of patient enrollment.

A total of 11,111 patients were diagnosed with CRC at our centers during the 12-year study period. Among those, 2,102 patients were classified as mCRC with known *KRAS* status. Of those, there were 1,130 patients (54%) with *KRASwt* mCRC. Among those with *KRASwt* data, 413 received anti-EGFR Ab as second- or later-line treatment, and those patients were included in this study. A Consolidated Standards of Reporting Trials (CONSORT) diagram of the patient enrollment process is shown in **Figure 1**.

### Patient Characteristics

The median age of included patients was 61 years (range: 29-93), and the ratio of males to females was 1.7:1. Seventy (17%) patients had right-sided tumors, and 343 (83%) patients had left-sided tumors. The liver and lung were the most frequent sites of metastasis. Two hundred and thirty-eight (58%) patients received anti-EGFR Ab in the third-line, and 132 (32%) patients and 43 (10%) patients were treated in the second- and the later-line, respectively. Single-agent irinotecan therapy was the most frequently used chemotherapy (73%) in combination with anti-EGFR Ab therapy. Patient and tumor characteristics and details of treatments according to tumor sidedness are shown in **Table 1**.

**Table 1:** Patient and tumor characteristics (N=413).

Characteristics			Right–sided		Left–sided		P–value
	n	%	n	%	n	%	
**Age, median (range)**	61 (29–93)		62 (29–83)		61 (29–93)		
**Gender**							
Female	154	37.3	31	44.3	123	35.9	0.18
Male	259	62.7	39	55.7	220	64.1	
**Metastatic site**							
Liver	319	77.2	53	75.7	266	77.6	0.74
Lung	175	42.4	34	48.6	141	41.1	0.25
Peritoneal	64	15.5	19	27.1	45	13.1	**0.003**
Non regional lymph nodes	97	23.5	20	28.6	77	22.4	0.27
Bone or other	43	10.4	8	11.4	35	10.2	0.76
**Metastatic type**							
Synchronous	251	60.8	42	60	209	60.9	0.88
Metachronous	162	39.2	28	40	134	39.1	
**Histology**							
Adenocarcinoma	397	96.1	65	92.9	332	96.8	0.45
Mucinous	9	2.2	3	4.3	6	1.7	
Signet–ring cell	7	1.7	2	2.9	5	1.5	
**Differentiation**							
Well	69	16.7	12	17.1	57	16.6	0.54
Moderately	277	67.1	46	65.7	231	67.3	
Poorly	30	7.3	8	11.4	22	6.4	
No data	37	8.9	4	5.7	33	9.6	
**MMR status**							
pMMR	68	16.5	12	17.1	56	16.3	**0.001**
dMMR	3	0.7	3	4.3	0	0	
No data	342	82.8	55	78.5	287	83.7	
**Line of treatment**							
Second–line	132	32	25	35.7	107	31.2	0.17
Third–line	238	57.6	42	60	196	57.1	
Later–line	43	10.4	3	4.3	40	11.7	
**Anti–EGFR Ab**							
Cetuximab	362	87.7	68	97.1	294	85.7	0.005
Panitumumab	51	12.3	2	2.9	49	14.3	
**Chemotherapy (+anti–EGFR Ab)**							
Capecitabine/5FU	1	0.2	1	1.4	0	0	0.17
FOLFOX/XELOX	21	5.1	3	4.3	18	5.2	
FOLFIRI/XELIRI	88	21.3	15	21.4	73	21.3	
Single irinotecan	303	73.4	51	72.9	252	73.5	

pMMR, proficient mismatch repair; dMMR, deficient mismatch repair; anti–EGFR Ab, anti–epidermal growth factor receptor antibodies; FOLFOX, combination leucovorin calcium (folinic acid), fluorouracil, and oxaliplatin; XELOX, combination capecitabine and oxaliplatin; FOLFIRI, combination leucovorin calcium (folinic acid), fluorouracil, and irinotecan hydrochloride; XELIRI, combination capecitabine and irinotecan.

A p–value < 0.05 indicates statistical significance. Bold means Statistically significant.

### *RAS* Testing Methods

Sixty-one percent (251/413) of patients had only *KRAS* exon 2 data, whereas 39% (162/413) of patients had extended *RAS* analysis data.

### Pattern of Response to Anti-EGFR Ab

An overall response rate (complete or partial response) was observed in 31.2% of the patients with left-sided tumor as compared with 18.6% in patients with right-sided tumor. There was no complete response. The percentage of patients with progressive disease was higher in the right-sided tumors group than in the left-sided tumors group (38.6% vs. 24.8%). Tumor response assessment was not available in 23 patients. Details of treatment response according to tumor sidedness are shown in **Table 2**.

**Table 2:** Pattern of Response to anti–EGFR Ab (N=413).

Pattern of response	N (%)	2^nd^–line		3^rd^–line		Later-line	
		Lt–sided	Rt–sided	Lt–sided	Rt–sided	Lt–sided	Rt–sided
CR	0	0	0	0	0	0	0
PR	120 (29.1)	32 (29.9)	7 (28)	63 (32.1)	6 (14.3)	12 (30)	0 (0)
SD	158 (38.3)	42 (39.3)	8 (32)	71 (36.2)	19 (45.2)	18 (45)	0 (0)
PD	112 (27.1)	26 (24.3)	10 (40)	51 (26.0)	14 (33.3)	8 (20)	3 (100)
NA	23 (5.6)	7 (6.5)	0 (0)	11 (5.6)	3 (7.1)	2 (5)	0 (0)
Total	413 (100)	107 (100)	25 (100)	196 (100)	42 (100)	40 (100)	3 (100)
		P=0.29		P=0.15		P=0.025	

CR, complete response; PR, partial response; SD, stable disease; PD, progressive disease; NA, not available.

A p–value < 0.05 indicates statistical significance.

### Reasons for Termination of Anti-EGFR Treatment

Disease progression was the most common cause of anti-EGFR therapy discontinuation followed by 6-month treatment completion of anti-EGFR therapy (51% and 36%, respectively). Eight percent of patients discontinued anti-EGFR Ab due to toxicities. Details of reason for termination of anti-EGFR treatment are shown in **Table 3**.

**Table 3:** Reasons for termination of anti–epidermal growth factor receptor (anti–EGFR) treatment (N=413).

Reasons	Total	Left–sided	Right–sided	*p*–value
	n (%)	n (%)	n (%)	
Disease progression	209 (50.6)	165 (48.1)	44 (62.9)	0.05
Completed 6 months	147 (35.6)	132 (38.5)	15 (21.4)	
Side effect	33 (8)	26 (7.6)	7 (10)	
Other	24 (5.8)	20 (5.8)	4 (5.7)	

A p–value < 0.05 indicates statistical significance.

### Survival Analysis

The median follow-up time was 28.7 months (mo). At the last follow-up visit (1 September 2021), there were 23 patients (5.6%) alive, and the remaining 390 patients (94.4%) had succumbed to their disease.

**Figure 2:**
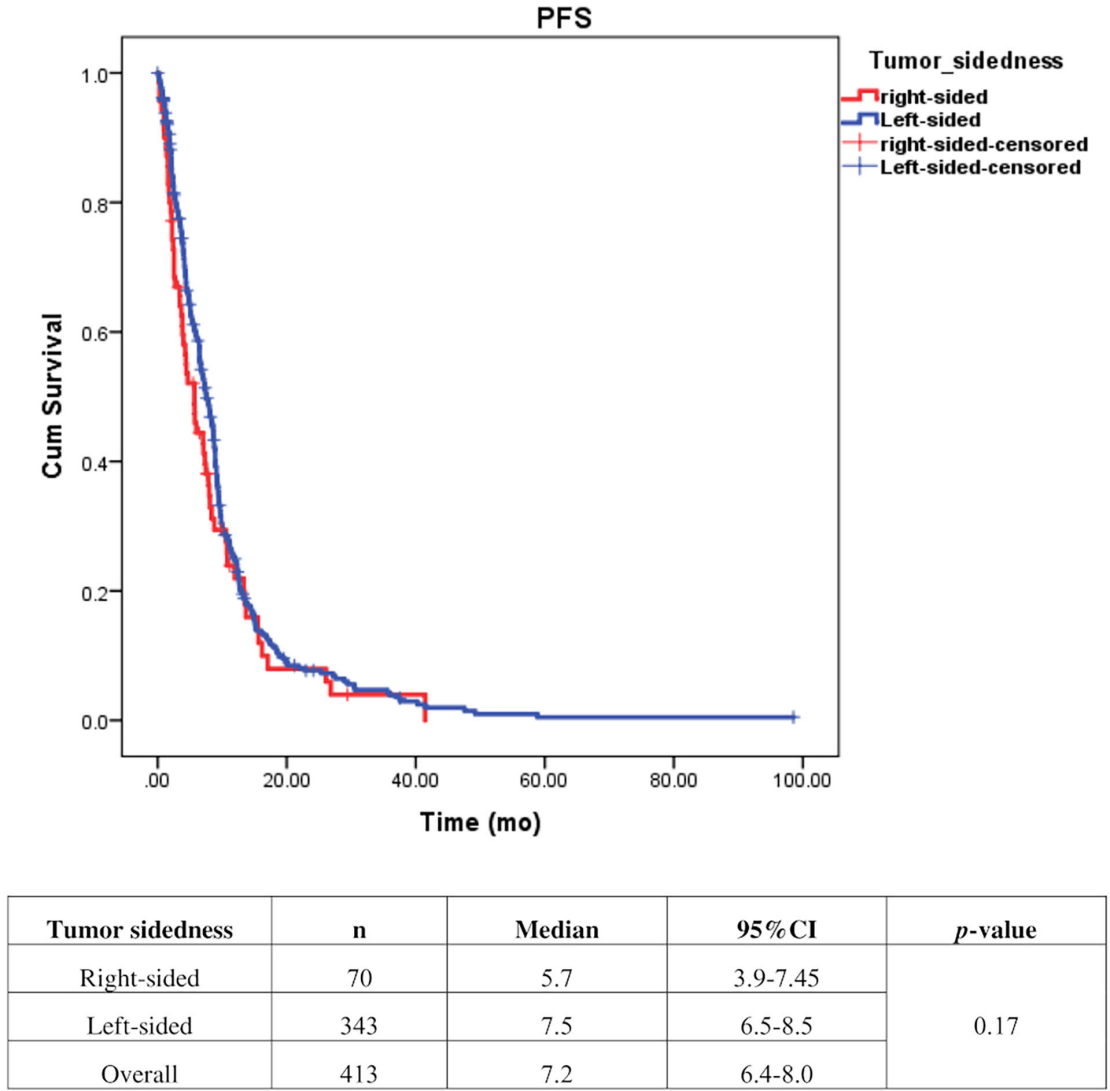
Effect of anti–EGFR treatment on PFS compared between right–sided tumor and left–sided tumor.

**Figure 3:**
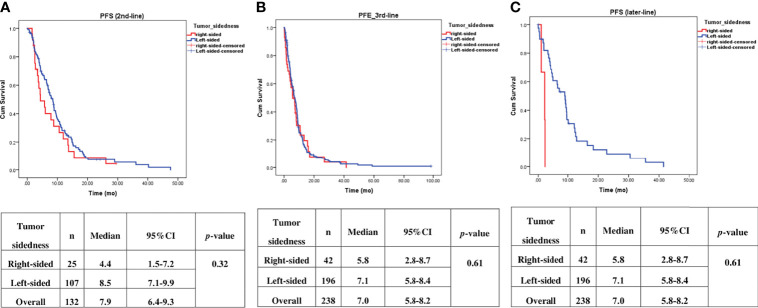
Effect of anti–EGFR treatment on PFS compared between right–sided tumor and left–sided tumor and stratified by treatment lines, **(A)** 2nd–line, **(B)** 3rd–line, **(C)** later–line.

**Figure 4:**
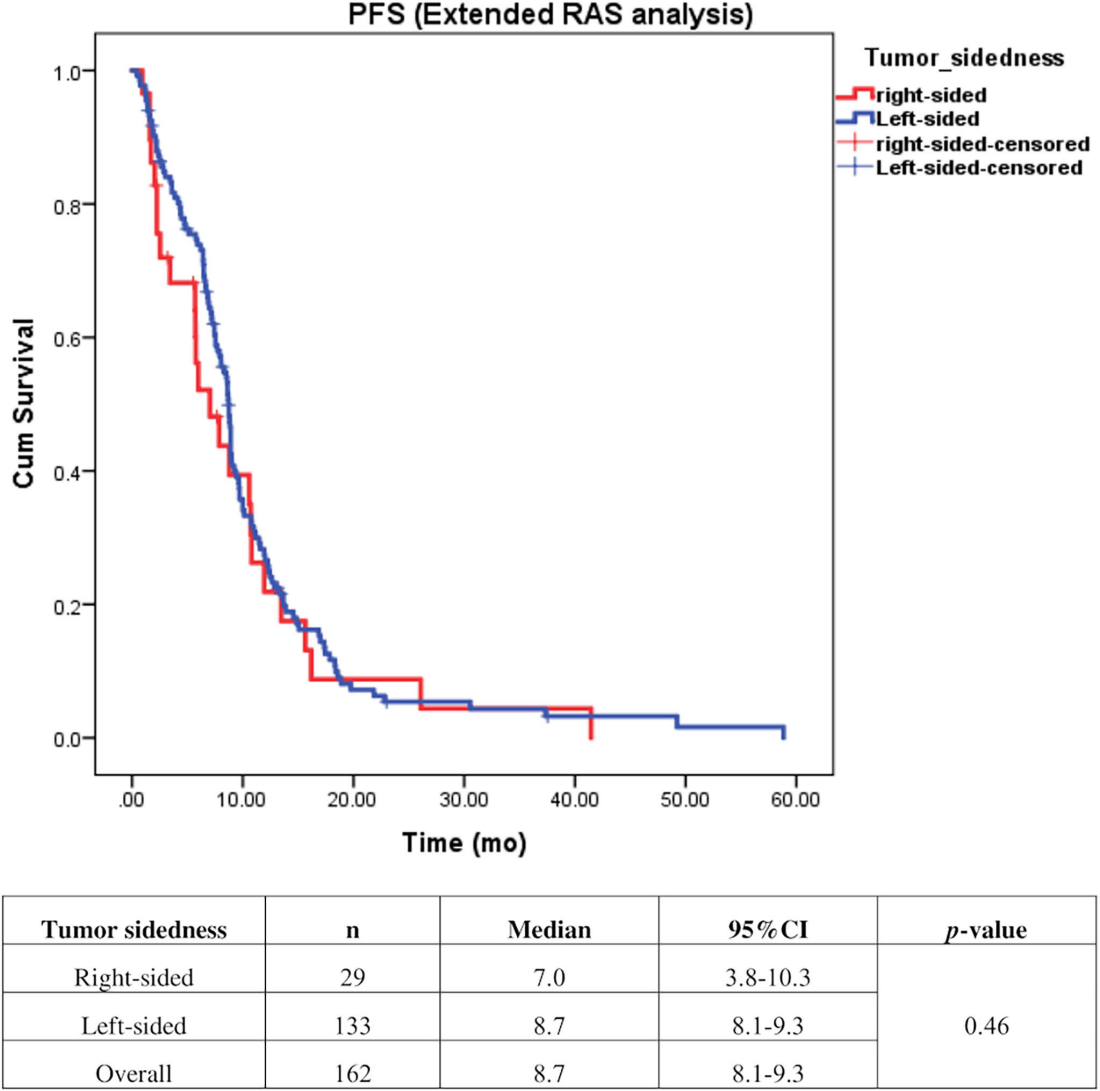
Effect of anti–EGFR treatment on PFS compared between right–sided tumor and left–sided tumor among patients who underwent extended *RAS* analysis (n=162).

**Figure 5:**
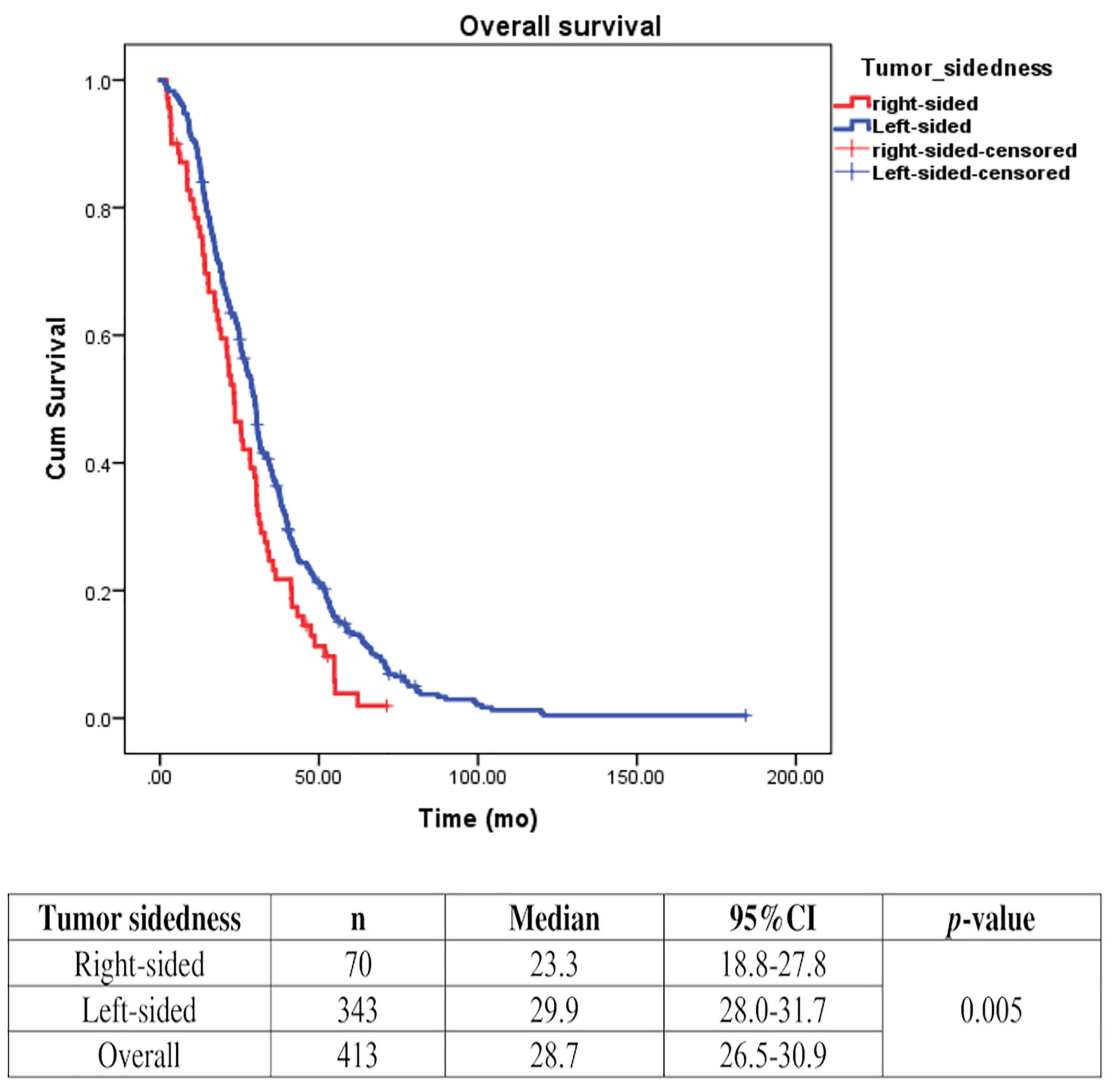
Effect of anti-EGFR treatment on OS compared between rightsided tumor and left-sided tumor.

Univariate analysis of PFS was performed using previously established prognostic factors. Factors that were associated with statistically significantly worse PFS in this analysis included mucinous/signet ring cell histology (p=0.02) and poorly differentiation (p=0.001). Patients with right-sided tumors had a non-significantly inferior PFS compared to those with left-sided tumors (median PFS: 5.7 mo, 95% CI: 3.9-7.5 *vs.* 7.5 mo, 95% CI: 6.5-8.5, respectively; *p*=0.17) (**Table 4** and **Figure 2**). Subgroup analysis of the impact of primary tumor location showed no significant difference in PFS between tumor locations when stratified by treatment lines except for later-line (**Figure 3**). Subgroup analysis of patients who had extended *RAS* analysis data showed no significant difference in PFS between the right-sided and left-sided groups (median PFS: 7.0 mo, 95% CI: 3.8-10.3 *vs.* 8.7 mo, 95% CI: 8.1-9.3, respectively; *p*=0.46) (**Figure 4**). Multivariate Cox proportional hazards regression analysis of PFS was performed using the factors mentioned above. In this analysis, moderately and poorly differentiated tumors were associated with worse PFS (HR 1.38, 95% CI 1.0-1.9; p=0.03 and HR 2.2, 95% CI 1.3-3.8; p=0.003, respectively) (**Table 4**). Among the entire cohort, patients with left-sided colon tumors had significantly better OS than those with right-sided tumors (median OS: 29.9 mo, 95% CI: 28.0-31.7 *vs.* 23.3 mo, 95% CI: 18.8-27.8, respectively; *p*=0.005) (**Figure 5**).

**Table 4:** Univariate and multivariate analysis on PFS for anti–EGFR Ab.

Variables	N	Univariate analysis			Multivariate analysis		
		Median survival (mo)	95%CI	P value	HR	95%CI	P value
**Gender**							
female	154	6.8	5.2–8.5	0.44			
male	259	7.4	6.2–8.6				
**Sidedness**							
Right–sided	70	5.7	3.9–7.5	0.17	Ref	0.6–1.1	0.29
Left–sided	343	7.5	6.5–8.5		0.86		
**Metastatic type**							
Synchronous	251	7.7	6.8–8.6	0.25	Ref	0.9–1.4	0.24
Metachronous	162	5.8	4.1–7.4		1.1		
**Histology**							
Adenocarcinoma	397	7.3	6.5–8.2	**0.02**	Ref	0.6–2.8	0.46
Mucinous	9	3.7	1.3–6.2		1.33	0.5–4.5	0.43
Signet ring cell	7	4.4	0.0–11.3		1.54		
**Differentiated**							
Well	69	9.4	7.9–10.9	**0.001**	Ref	1.0–1.9	**0.03**
Moderately	277	7.1	6.2–8.0		1.38	1.3–3.8	**0.003**
Poorly	30	3.8	1.8–5.9		2.2		
**MMR status**							
pMMR	68	28.8	8.8–9.5	0.35			
dMMR	3	2.5	1.1–4.0				
**Line of treatment**							
Second–line	132	7.8	6.4–9.3	0.56	Ref	0.9–1.5	0.25
Third–line	238	7.0	5.8–8.2		1.2	0.8–1.7	0.43
Later–line	43	7.2	6.4–8.0		1.2		

pMMR, proficient mismatch repair; dMMR, deficient mismatch repair; anti–EGFR Ab, anti–epidermal growth factor receptor antibodies.

A p–value < 0.05 indicates statistical significance. Bold means Statistically significant.

## Discussion

The results of this study demonstrated that tumor sidedness has no significant impact on treatment outcomes in *KRASwt* mCRC patients treated with anti-EGFR Ab in second- or later-line treatment. We also confirmed that patients with right sided tumors had worse outcome.

Metastatic CRC is a genetically heterogeneous disease with tumors arising from different sides of the colon (left versus right), and having different clinical and molecular characteristics (9). The incidence rates of left- and right-sided CRC also differ markedly with approximately two-thirds of CRC developing on the left side, and the remaining one-third developing on the right side (10).

Primary tumor location plays a significant role in estimating prognosis in mCRC. A retrospective analysis of CALGB/SWOG80405 (11) revealed that patients with *KRASwt* (codons 12 and 13) mCRC had significantly prolonged median OS in patients with left-sided tumors when compared to those with right-sided tumors irrespective of allocation to the cetuximab or bevacizumab groups. A recent retrospective analysis of six randomized trials that included 2,159 unresectable *RASwt* mCRC patients also found a worse prognosis for OS, PFS, and ORR in patients with right-sided tumors compared with those with left-sided tumors (12). These findings were consistent with the results from two recent meta-analyses (13, 14). Our study also demonstrated that patients with right-sided *KRASwt* tumors had significantly worse outcome (median OS: 23.3 mo and 29.9 mo for right-sided tumors and left-sided tumors, respectively (*p*=0.005). Therefore, our data confirmed that tumor sidedness does have prognostic impact on survival outcome in mCRC.

Regarding the predictive value of primary tumor location, CALGB/SWOG80405 (11) reported the median OS with cetuximab-based therapy to be 37.5 mo in left sided tumors as compared to 32.1 mo with bevacizumab-based therapy (HR: 0.77, *p*<0.05). For the right sided tumors, the bevacizumab arm had an OS of 24.5 mo compared to 16.4 mo in the cetuximab arm. Arnold, et al. (12) published the results of a retrospective pooled analysis of six randomized studies of tumor sidedness and anti-EGFR therapy in patients with *RASwt* (*KRAS/NRASwt*) mCRC. Of those six trials, five trials were first-line therapy [CRYSTAL (15), FIRE-3 (5), PRIME (16), PEAK (17), and CALGB/SWOG 80405 (2)]. In patients with left-sided tumors, doublet chemotherapy (either FOLFOX or FOLFIRI) plus anti-EGFR Ab (either cetuximab or panitumumab) was found to be associated with improved survival compared with doublet chemotherapy with or without bevacizumab (HR for OS: 0.75 and HR for PFS: 0.78). However, no benefit of anti-EGFR therapy was observed in patients with right-sided mCRC (HR for OS: 1.12 and HR for PFS: 1.12). Holch, et al. (14) performed a meta-analysis of 13 first-line randomized controlled trials and one pharmacogenetic study. Pooled data from all first-line anti-EGFR *vs.* anti-VEGF studies in *RASwt* mCRC patients (CALGB/SWOG 80405, FIRE-3, PEAK) showed significant OS benefit of anti-EGFR therapy in left-sided tumors (HR: 0.71, *p*=0.0003). Non-significant OS benefit favoring anti-VEGF (HR: 1.3, *p*=0.081) in patients with right-sided tumors was observed.

From these data, the ESMO consensus guidelines (7) recommend adding anti-EGFR Ab as a first-line treatment only in patients with left-sided *RAS*wt mCRC. These findings are now incorporated into the latest clinical practice guidelines in European countries. However, the National Comprehensive Cancer Network (NCCN) still has no clear preference or recommendation relative to the addition of a biological agent in patients with left-sided tumors (18).

Although there are published recommendations specific to first-line treatment in mCRC, there are no recommendations relative to tumor sidedness in second- and subsequent-line treatment. The NCCN panel stated that there is not enough evidence to include tumor sidedness in treatment selection in these settings (18). To date, only few data have been reported specific to the effect of primary tumor location on the outcomes for *RAS*wt patients receiving second- or later-line anti-EGFR treatment.

A retrospective analysis by Boeckx, et al. (19) evaluating *RASwt* data from 2 randomized studies [study 20050181 (20), and 20020408 (21)] revealed that *RAS*wt left-sided tumors had clinical benefit when panitumumab was added in the second- or later-line treatment. In study 20050181, the addition of panitumumab to FOLFIRI resulted in a numerically improved median OS (20.1 mo *vs.* 16.6 mo; HR: 0.96; *p*=0.74) and PFS (8.0 mo *vs.* 5.8 mo; HR: 0.88; *p*=0.31) when compared with FOLFIRI alone in patients with *RASwt* left-sided primary tumors. In right-sided tumors, the HR for PFS favored panitumumab (4.8 mo *vs.* 2.4 mo; HR: 0.75; *p*=0.29), but the HR for OS favored FOLFIRI alone (10.3 mo *vs.* 8.1 mo; HR: 1.14; *p*=0.62). In study 20020408, a significant PFS benefit (5.5 mo *vs.* 1.6 mo; HR: 0.31; *p*<0.0001) was observed when panitumumab was added to best supportive care (BSC) to treat *RASwt* left-sided mCRC patients. No significant difference in PFS was observed in patients with right-sided tumors (1.7 mo *vs.* 1.5 mo; HR: 0.50; *p*=0.10). The OS results in that study were difficult to interpret because most patients in the BSC arm crossed over to panitumumab at progression.

Our finding demonstrated that patients with right-sided tumors had a non-significantly inferior PFS compared to those with left-sided tumors (median PFS: 5.7 mo, *vs.* 7.5 mo, *p*=0.17). Subgroup analysis also showed no significant difference in PFS between left-or right-sided when stratified by treatment lines (*p*=0.32 and 0.61 in second-and third-line, respectively). Due to small number of patients in later-line having right-sided tumors, no conclusion can be drawn from our cohort. A previous retrospective study reported that a *KRAS* exon 2 mutation alone cannot fully explain the heterogeneity of treatment responses to anti-EGFR therapy in mCRC (3). Strong evidence suggests that extended *RAS* analysis is needed to facilitate the identification of patients who most likely to benefit from anti-EGFR therapy. We then analyzed the patients who had extended *RAS* analysis data, and we found no significant difference in PFS between left- and right-sided tumors (median PFS: 7 mo for right-sided tumors, and 8.7 mo for left-sided tumors; *p*=0.46), which confirmed that sidedness had no significant impact on subsequent lines of treatment. Therefore, there was insufficient evidence to support the use of tumor sidedness in treatment selection in second- or later-line.

In this study, we did not directly compare the outcome in patients receiving chemotherapy with or without anti-EGFR Ab, since the majority of our patients were treated in the third-line setting. It might not be justified to compare with regorafenib or TAS102 which is considered to be the standard third-line therapy. Therefore, the predictive impact of sidedness for benefit of adding anti-EGFR Ab to chemotherapy in later-line treatment cannot be elucidated.

In our study, the ORR was approximately 30% in patients with left-sided tumors across treatment lines. For right-sided tumor, the ORR was 28% and 14% in second- and third-line respectively. Since the ORR was only 1-3% in patients who were treated with regorafenib (22, 23) or TAS102 (24, 25) in third- or later-line setting, our data supported the use of anti-EGFR Ab plus irinotecan in third-line treatment in patients with no prior anti-EGFR Ab before regorafenib or TAS102 regardless of tumor sidedness.

### Study Strengths and Limitations

The strength of this study is a multi-center study which reduces the risk of bias. We also included patients with extended *RAS* analysis, which reflected the current guideline for using anti-EGFR Ab. However, owing to retrospective nature of the study, there are some inherent limitations. Firstly, some patients had missing or incomplete data. Secondly, due to relatively small number of right-sided tumors, the statistical power of the subgroup analysis might be insufficient for identifying all significant differences and should be interpreted with caution. Lastly, considering that the health benefit scheme in Thailand limits for only 6-month coverage for anti-EGFR Ab treatment, one-third of patients had to stop anti-EGFR Ab after 6-month treatment, even in cases that were responding to treatment. This factor could confound the duration of PFS. Nevertheless, our study is one of the few real-world studies that specifically addressed the effect of primary tumor location on the clinical outcomes of patients with *KRASwt* mCRC that were treated with anti-EGFR Ab therapy as second- or later-line treatment.

## Conclusion

To date, this is the largest real world data of the effect of primary tumor location on anti-EGFR Ab which demonstrated that tumor sidedness has no significant impact on treatment outcomes in *KRASwt* mCRC patients receiving second- or later-line therapy. Our findings do not support the utility of tumor sidedness for treatment selection in these settings. We confirmed that patients with right-sided tumors had significantly worse survival.

## Data Availability Statement

The original contributions presented in the study are included in the article/supplementary material. Further inquiries can be directed to the corresponding author.

## Ethics Statement

This retrospective nature of this study ensures total anonymity of patient data, and patient health and wellbeing are in no way affected, so the requirement to obtain written informed consent from study participants was waived.

## Author Contributions

Conception and design: KK. Administrative support: KK. Provision of study materials or patients: AA, NT, TS, NP, ST, ES, CA, and KK. Collection and assembly of data: KK, AA, NT, and TS. Data analysis and interpretation: KK. Manuscript writing: all authors. Final approval of manuscript: all authors. All authors contributed to the article and approved the submitted version.

## Conflict of Interest

The authors declare that the research was conducted in the absence of any commercial or financial relationships that could be construed as a potential conflict of interest.

## Publisher’s Note

All claims expressed in this article are solely those of the authors and do not necessarily represent those of their affiliated organizations, or those of the publisher, the editors and the reviewers. Any product that may be evaluated in this article, or claim that may be made by its manufacturer, is not guaranteed or endorsed by the publisher.
